# Biomaterial-enabled multimodal therapy for endometrial cancer: Subtype-guided immuno-metabolic modulation and fertility-conscious regeneration

**DOI:** 10.1016/j.mtbio.2026.103121

**Published:** 2026-04-18

**Authors:** Min Wang, Cuihong Wang, Bing Xin, Lijie Zhao, Benshuo Cai, Yuheng Guan, Lu Lu, Chong Zhang

**Affiliations:** a Department of Obstetrics and Gynecology, Shengjing Hospital of China Medical University, Shenyang, Liaoning, China; b Department of Pulmonary and Critical Care Medicine, Shengjing Hospital of China Medical University, Shenyang, Liaoning, China; c Department of Interventional Therapy, The First Hospital of China Medical University, Shenyang, Liaoning, China; d Department of Ophthalmology, The Fourth Affiliated Hospital of China Medical University, Eye Hospital of China Medical University, Key Lens Research Laboratory of Liaoning Province, Shenyang, Liaoning, China; e Department of Infectious Disease, Shengjing Hospital of China Medical University, Shenyang, Liaoning, China; f The Rogel Cancer Center, Department of Internal Medicine, University of Michigan, Ann Arbor, MI, 48109, United States

**Keywords:** Endometrial cancer, Subtype-guided therapy, Biomaterial-enabled drug delivery, Immuno-metabolic reprogramming, Fertility preservation

## Abstract

Endometrial cancer (EC) encompasses various histological and molecular subtypes, each with unique immune, endocrine and metabolic characteristics, resulting in varying responses to treatment. Although immunotherapy, endocrine therapy and metabolic targeted therapy have broadened treatment options, the clinical efficacy varies due to subtype-specific drug resistance, systemic side effects, and the need for fertility preservation in young patients. This requires us to shift from simple combination therapy to a treatment model that integrates biological and subtype-specific information. The latest advancements in the field of biomaterials provide new approaches to achieve this integration. Tools such as nanocarriers and hydrogels can enable precise and localized drug delivery in the uterus, achieving controlled release based on specific tumor biological characteristics. For tumors with immune activity, they can locally deliver immune stimulants; for certain subtypes, they can directly provide continuous endocrine therapy within the tissue, avoiding systemic exposure. In cases of resistance, they can modify local metabolism to restore treatment sensitivity. Essentially, biomaterials act as intelligent interfaces, converting subtype-specific information into targeted treatment strategies. Specifically, biomaterial platforms can utilize endogenous tumor and tissue cues (pH, enzyme activity, redox state, or inflammatory signals) to trigger the on-demand release of immunomodulators, endocrine drugs, or metabolic regulators in the uterine microenvironment, thereby enhancing local efficacy while reducing systemic toxicity. Importantly, biomaterial-enabled strategies extend beyond tumor control by incorporating endometrial repair and regeneration into therapeutic design. By temporally decoupling antitumor intervention from regenerative signaling within a single platform, these approaches directly address the fertility-preserving imperative of EC management. This review systematically presents new multimodal therapeutic strategies for EC made possible by biomaterials, focusing on how spatial, temporal and biological programmability enables subtyped treatment, immunometabolic reprogramming and integrated regeneration. We explore design principles, translational challenges and clinical implications, proposing biomaterials as a paradigm for precision therapy focused on fertility preservation. It is important to note that this review systematically positions endometrial regeneration focused on preserving fertility as a fundamental therapeutic principle rather than as a complement downstream of tumor control, advocating its integration into comprehensive treatment plans from the start of subtyp-targeted therapy.

## Graphical abstract

**Figure:**
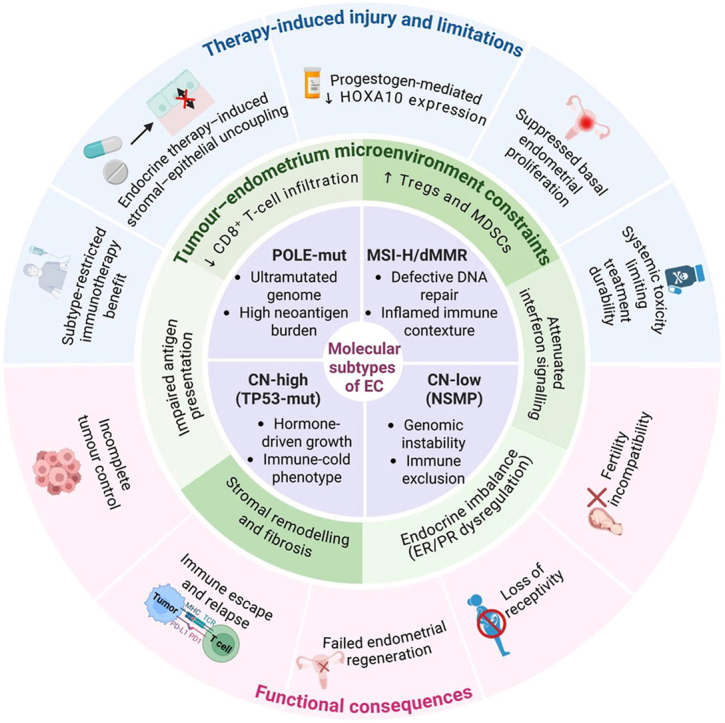

## 1 Introduction

### 1.1 Clinical landscape and unmet needs in endometrial cancer

**Fig. 1:**
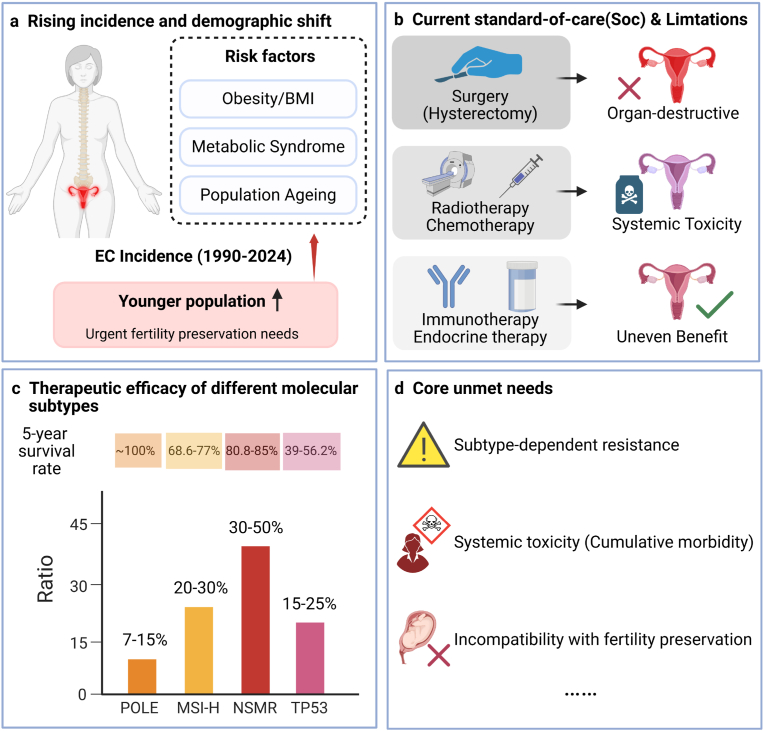
**Clinical heterogeneity and unmet therapeutic needs in EC.** The incidence of EC is continuously increasing worldwide. The driving factors include population aging, obesity, and metabolic disorders, and the proportion of diagnoses among young women is increasing. Although most patients are diagnosed at an early stage and receive successful short-term prognosis after surgical treatment, a considerable proportion of patients experience recurrence or progression, and the prognosis deteriorates sharply in advanced or recurrent cases. The current standard treatment strategies, including hysterectomy, radiotherapy, systemic chemotherapy, endocrine therapy, and ICB, have significant differences in efficacy, and are often accompanied by cumulative toxicity. It is notable that there is a high degree of imbalance in the therapeutic benefits of different molecular subtypes: endocrine therapy only produces incomplete and often short-lived responses for hormone-driven tumors; chemotherapy can only provide moderate and short-term disease control; while immunotherapy mainly has significant benefits for mismatch repair deficiency or POLE mutation types of diseases, and has limited efficacy for most ECs. These patterns collectively highlight the convergence of unmet needs, including subtype-dependent treatment resistance, systemic toxicity, and the incompatibility of traditional treatments with fertility preservation. To address these challenges, it is essential to go beyond a simple tumor eradication treatment model and integrate biological accuracy, persistence and retention of uterine function.

Endometrial cancer (EC) is the most common malignancy of the female reproductive tract in developed countries [[1], [2], [3]]^,^ with a steadily rising incidence driven by population ageing, obesity and metabolic disorders (Fig. 1) [4,5]. Although approximately 70-80% of patients are diagnosed with early-stage disease and achieve favorable short-term outcomes following surgery-based management, recurrence and treatment failure remain clinically significant. Up to 20-30% of patients with ostensibly early-stage EC ultimately experience relapse, and survival outcomes decline sharply in advanced or recurrent disease, where five-year overall survival may fall below 20% [[6], [7], [8]] [.

Current treatment paradigms rely heavily on hysterectomy with bilateral salpingo-oophorectomy, often followed by adjuvant radiotherapy or systemic chemotherapy in higher-risk settings [9,10]. While effective for local tumor control, these approaches are intrinsically organ-destructive and incompatible with fertility preservation. This limitation is increasingly consequential as EC is diagnosed with growing frequency in younger women, for whom long-term quality of life and reproductive potential are central clinical considerations [11,12]. Meanwhile, advances in diagnostic imaging and increased awareness have led to more frequent detection of early-stage disease, providing a window of opportunity for fertility-sparing management in selected patients [13].

Fertility-sparing strategies based on prolonged endocrine therapy have therefore emerged as an alternative for carefully selected patients with low-grade, hormone-responsive tumors [14]. However, clinical efficacy is limited. Complete response rates of approximately 60-80% are achieved in optimal candidates, yet recurrence rates approach 30-40% during long-term follow-up. Moreover, durability is frequently undermined by endocrine resistance driven by compensatory activation of oncogenic signaling pathways, most notably the PI3K-AKT-mTOR axis, as well as by metabolic adaptation. These limitations highlight the fragility of endocrine monotherapy as a long-term solution. Systemic chemotherapy remains the cornerstone of treatment for advanced and high-risk EC, typically using a platinum-taxane combination regimen. However, its efficacy is limited and short-lived, with the objective response rate for recurrent disease typically being only 30%-40%, and the median progression-free survival rarely exceeding one year. Cumulative toxicity limits the dosage and duration of treatment, and has a particularly significant impact on younger patients, further complicating the management of fertility awareness.

The emergence of immunotherapy has reshaped the treatment landscape for EC, while also revealing significant biological inequalities in treatment benefits among different molecular subtypes [15]. Tumors with mismatch repair deficiency (MMR-d) or microsatellite instability high (MSI-H) status, accounting for approximately 25%-30% of EC, exhibit significant sensitivity to immune checkpoint inhibition, with an objective response rate (ORR) of up to 40%-50% in advanced or recurrent disease. In contrast, most EC, including low-copy number (CN-low) tumors (without specific molecular features, NSMP) and high-copy number (CN-high) tumors (TP53-mutant)) show significantly reduced benefits, even with combined treatment regimens, and their response rates typically remain below 10%-15%. These differences highlight the persistent presence of immunosuppressive tumor microenvironments in non-immunogenic subtypes and indicate the limited translational potential of immunotherapy in a broader population of EC patients.

Multiple key clinical trial data support the subtype-specific activity of immune checkpoint inhibitors (ICIs) in EC. In the Phase II Keynote-158 study (NCT02628067), 90 patients with advanced MSI-H/dMMR endometrial cancer who had previously received treatment were treated with pembrolizumab (200 mg every 3 weeks) for up to 35 cycles [16]. Among 79 efficacy-evaluable patients, the ORR was 48% (95% CI, 37-60), including complete responses in 14% and partial responses in 34%. Median progression-free survival (PFS) was 13.1 months, and median overall survival (OS) was not reached after a median follow-up of 42.6 months. Treatment-related grade 3-4 adverse events occurred in approximately 12% of patients, indicating a favorable therapeutic index in this biologically defined subgroup. Consistent results were observed in the phase I GARNET trial evaluating dostarlimab (NCT02715284), which enrolled 143 patients with dMMR/MSI-H EC and 156 patients with mismatch repair-proficient/microsatellite-stable (MMRp/MSS) disease [17]. ORRs of ∼43.5% were achieved in the dMMR/MSI-H cohort, whereas responses in the MMRp/MSS group were markedly lower (∼14.1%), despite evidence of durable responses and manageable toxicity profiles. Together, these data reinforce the conclusion that intrinsic tumor immunogenicity is a dominant determinant of ICI responsiveness in EC.

For the large proportion of patients with immunologically cold tumors, combination strategies have been explored to broaden therapeutic benefit [18,19]. In KEYNOTE-775, combined PD-1 and VEGFR inhibition with pembrolizumab plus lenvatinib significantly improved PFS (hazard ratio ∼0.60) and OS compared with chemotherapy in advanced EC, including patients with pMMR disease. These findings have informed current treatment guidelines and illustrate that microenvironmental modulation can partially overcome primary immune resistance. However, the remission rate remains low and the treatment-related toxicity is significant, highlighting the incompleteness of this approach. These results collectively reveal a fundamental limitation in the current management of EC treatment: treatment decisions are still mainly based on anatomical staging and histopathological classification, rather than molecular subtypes and the microenvironmental background [20]. Therefore, many patients receive treatment regimens that are biologically mismatched, have insufficient efficacy, or are accompanied by unacceptable systemic toxicities. Moreover, standard treatment regimens rarely consider fertility preservation or the integrity of the endometrium, which further limits the improvement of long-term quality of life.

**Scheme 1:**
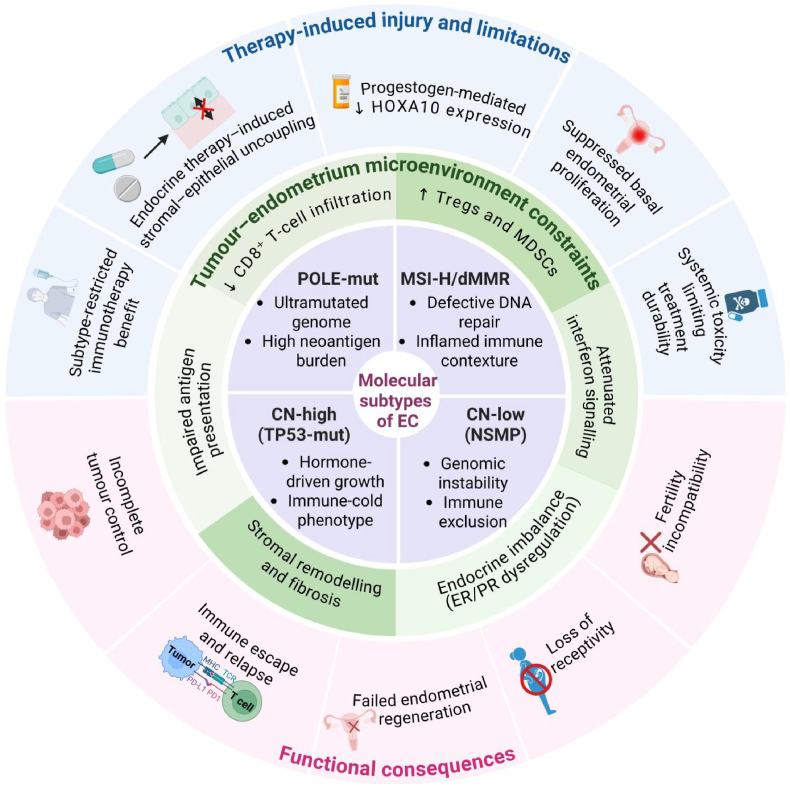
A multilayer framework integrating tumor control and endometrial regeneration in EC. The therapeutic response and regenerative capacity of EC are governed by hierarchical and interdependent biological limitations. At its core, molecular subtypes (POLE-mutant type, MSI-H/MMR deficient type, CN-low type, and CN-high type) determine the intrinsic immunogenicity, genomic stability, and endocrine dependence of the tumor. These characteristics shape the tumor-endometrium microenvironment, which is characterized by variable immune infiltration, matrix remodeling, and endocrine signal balance. Standard therapies (including ICB and endocrine therapy) function under these limitations and may introduce additional tissue damage, such as decoupling of stroma-epithelium and downregulation of endometrial receptivity programs (such as HOXA10). The interaction between the intrinsic biological characteristics of tumors, the inhibition of the microenvironment, and the damage induced by treatment leads to incomplete tumor control and failure of endometrial regeneration, thereby limiting fertility preservation. Biomaterial-enabled strategies constitute engineered outer structures that can achieve spatiotemporal coordinated delivery of subtype-matched therapeutic drugs, local immune reprogramming, and extracellular matrix-guided repair, providing a framework for coordinating tumor therapeutic efficacy with uterine function recovery.

At present, EC is confronted with common challenges such as treatment resistance, systemic toxicity and impact on reproductive function. Therefore, the treatment concept must be upgraded: from solely pursuing tumor eradication to integrating precise biological therapy with preservation and reconstruction of uterine function. To achieve this goal, a new treatment strategy that can adapt to the molecular diversity of tumors needs to be constructed Scheme 1.

### 1.2 Molecular and biological heterogeneity as a determinant of therapeutic response

**Fig. 2:**
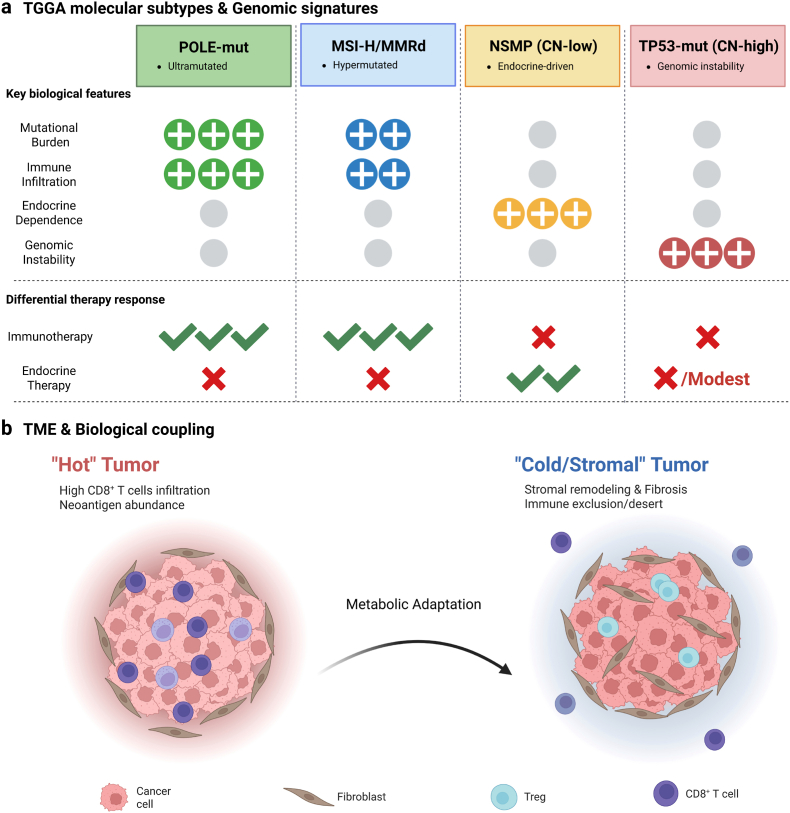
**Molecular heterogeneity of EC provides a biological basis for subtype-oriented multimodal treatment, as each subtype imposes unique immunological, endocrine and metabolic constraints, thereby affecting treatment sensitivity.** Integrated genomic analysis identified four major molecular subtypes of endometry cancer. POLE mutant subtype, MMRd/MSI-H subtype, CN-low expression subtype (no specific molecular signature, NSMP) and CN-high expression subtype (TP53 mutant subtype). These subtypes represent biologically distinct disease entities and not discrete points on a continuous line. Subtypes differ considerably in terms of mutation load, immune infiltration, endocrine dependence and genomic stability, resulting in varying therapeutic sensitivities. The mutation subtype pole showed high antigenic load and immune activation with MSI-H/MMRd tumors, responding significantly to ICB treatment; Whereas CN-low expression tumors are generally motivated by estrogen signaling and are initially sensitive to endocrine therapy, but are susceptible to develop resistance through compensated growth and metabolic pathways; CN-high expression tumors exhibit aggressive behaviour and limited response to most systemic treatments. Molecular subtypes also shape tumor TME, influencing immunosuppression, interstitial remodeling and metabolic adaptation. These interconnected characteristics highlight why unified treatment strategies can produce inconsistent effectiveness and underscore the need for a subtypy-oriented treatment framework that can harmonize multiple biological axes.

Since EC is not a single disease but consists of a series of histological and molecular subtypes with diverse biological behaviors and treatment sensitivities (Fig. 2), the traditional histopathological classification, such as differentiating endometrioid adenocarcinoma from non-endometrioid adenocarcinoma, has been unable to fully reflect its complexity and has limited guidance for treatment decisions. Integrative genomic analysis, especially the research of the Cancer Genome Atlas (TCGA), has established four main molecular subtypes: POLE mutant type, driven by pathogenic mutations in the DNA polymerase correction domain, characterized by a very high mutation load; mismatch repair deficiency type (MMR-d), often associated with high microsatellite instability; CN-low type, usually driven by estrogen, with a relatively stable genome; and CN-highr type, often presenting extensive chromosomal instability and TP53 gene mutation [[21], [22], [23], [24]]. hese subtypes constitute distinct biological entities, rather than different states on the same continuous spectrum [25,26].

This molecular-level heterogeneity has a direct and significant clinical impact on treatment [27,28]. For instance, MMR-d/MSI-H tumors typically have a high load of neoantigens and strong immunogenicity, and respond well to immune checkpoint inhibitors (ICB). In contrast, low-copy-number tumors are mostly driven by hormonal signaling pathways and are more sensitive to endocrine therapy, but they may also develop endocrine resistance due to compensatory growth and activation of metabolic pathways. High-copy-number tumors often have TP53 mutations, are highly invasive, and have a poor prognosis, and do not respond well to either immunotherapy or hormone therapy. The differences in subtype-specific treatment sensitivity explain, to some extent, why the previous unified treatment strategies have been difficult to achieve consistent efficacy in EC patients [29].

The key point is that the molecular subtypes not only affect the intrinsic signaling pathways of the tumor, but also regulate the composition and function of the tumor microenvironment (TME). Differences in immune cell infiltration, matrix remodeling, and metabolic flux are all closely related to genomic subtypes, jointly shaping different immune suppression patterns, endocrine feedback mechanisms, and metabolic adaptation pathways. Therefore, the treatment resistance of EC stems from the interrelated biological networks rather than isolated single pathways, and this cannot be solved by a single intervention method. This complexity highlights the necessity of establishing a new therapeutic framework: this framework should be based on molecular subtypes and be able to synergistically regulate multiple biological dimensions to achieve precise treatment.

### 1.3 Rationale for biomaterial-enabled integration of multimodal therapies

Immunotherapy, endocrine therapy, and metabolic targeted therapy have independently expanded the treatment options for EC. However, their clinical potential is limited by systemic toxicity, limited tumor selectivity, and the lack of coordinated temporal and spatial control. These limitations have led to an increasing focus on biomaterials as an enabling platform for therapeutic integration, rather than merely as delivery vehicles [30].

Breakthroughs in the field of biomaterial engineering - including nano-carriers, injectable hydrogels, and biomimetic scaffolds - now enable precise control over drug localization, release kinetics, and microenvironmental regulation. These platforms can concentrate therapeutic drugs in the uterine cavity or TME, minimizing off-target exposure and coordinating sequential or synergistic effects of different treatment modalities. Particularly important is that systems based on biomaterials can be rationally designed to match molecular subtype-specific biological characteristics, thereby activating immune responses in immunogenic tumors, enhancing endocrine sensitivity in hormone-driven diseases, or achieving metabolic reprogramming in treatment scenarios of drug resistance. Beyond tumor control, biomaterials uniquely enable the convergence of oncologic therapy and tissue regeneration. By supporting endometrial repair through structural scaffolding, bioactive signaling or stem cell delivery, these platforms offer a pathway towards fertility-conscious management that integrates treatment efficacy with restoration of uterine function [31]. This dual capability positions biomaterial-enabled multimodal therapy as a conceptual bridge between precision oncology and regenerative medicine in EC.

In this Review, we synthesize emerging evidence on biomaterial-enabled multimodal strategies for EC, emphasizing subtype-guided therapeutic logic, immuno-metabolic crosstalk and translational feasibility. By framing EC treatment through the integrated lenses of tumor biology and reproductive preservation, we propose a paradigm that moves beyond additive combination therapy towards biologically coordinated and clinically actionable precision care.

## 2 Subtype-guided logic of multimodal therapy: why integration is required, what to target and how to combine

The clinical heterogeneity of EC demands therapeutic strategies that are not only multimodal but also biologically coordinated. Simply layering immunotherapy, endocrine therapy and metabolic interventions in parallel has yielded limited success, largely because these modalities interact through shared signaling networks and are differentially constrained by molecular subtype. A subtype-guided framework is therefore essential to determine why multimodal integration is necessary, what biological axes should be prioritised, and how therapies can be combined in a rational and clinically actionable manner (Fig. 2) [29].

### 2.1 Biomaterials as organizers of multimodal therapy rather than passive carriers

Conventional drug delivery approaches treat biomaterials primarily as vehicles to improve solubility or pharmacokinetics. However, in the context of EC (peripheral vascular) diseases, this viewpoint seriously underestimates its therapeutic potential. The modern biomaterial platform, including nanoscale carriers [[32], [33], [34]], injectable hydrogels [35,36] and bioinspired matrices, these materials can actively regulate the spatial and temporal distribution of the treatment, thereby playing the role of a multi-modal therapeutic organizer rather than a passive delivery tool.

Spatial control is particularly crucial in the treatment of EC as the uterus provides a unique anatomical space that facilitates local intervention. Biomaterial-assisted localization techniques can achieve high drug concentrations within the tumor or within the uterus, while minimizing systemic exposure, this is crucial for immunomodulators and endocrine therapy as these drugs are often dose-limited due to off-target toxicity. Time control further supports sequential or phased treatment strategies, such as activating the TME before immune activation, or transitioning from the tumor suppression stage to the tissue repair stage.

It is important that biomaterials provide a means to coordinate the previously independent biological processes in treatment. By regulating drug release kinetics, co-delivery ratios, and microenvironmental signals, the biomaterial platform can achieve synergistic effects of immunomodulation, hormone signaling, and metabolic reprogramming within a single treatment framework. This organizational ability provides a structural basis for the construction of subtype-oriented multimodal therapies.

### 2.2 Immunotherapy in EC: defining its role through subtype-specific immune constraints

**Fig. 3:**
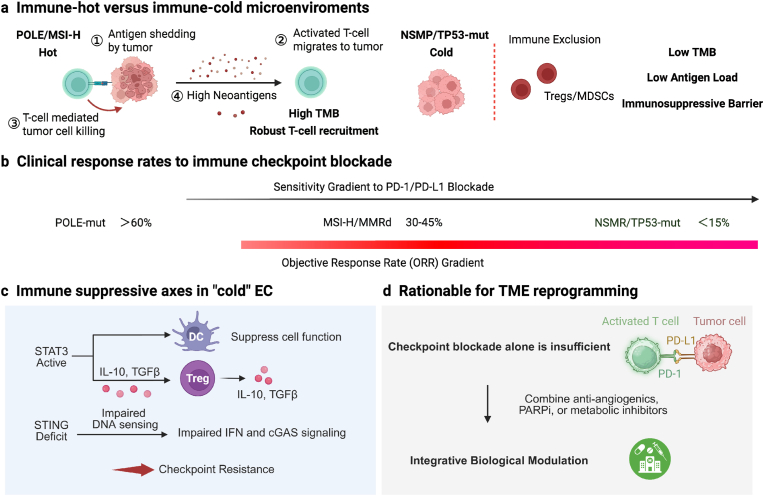
**Immunotherapy responsiveness of EC depends on subtype-specific immune restriction rather than simply ICB. This highlights the need for bio-material-mediated reprogramming of the microenvironment in immunologically cold diseases.** The clinical efficacy of ICIs in EC significantly depends on the molecular subtype. Tumors carrying pathogenic mutations in the exonuclease domain of DNA polymerase ε (POLE mutations) exhibit extremely high tumor mutational burden, dense infiltration of cytotoxic T cells, and abnormal sensitivity to PD-1/PD-L1 blockade, with an objective response rate typically exceeding 50%-60%. Mismatch repair-deficient or microsatellite instability-high expressing tumors account for approximately one quarter of EC cases, and although the treatment responses are consistent, the degree is relatively mild. Multiple clinical trials have shown an objective response rate of approximately 30-45%. In contrast, tumors with CN-low and CN-high subtypes exhibit limited immunogenicity, sparse immune infiltration, and dominant immunosuppressive networks. Even with combined treatment regimens, their response rates are typically lower than 10-15%. These response gradients reflect the fundamental differences in the landscape of neoantigens, immune architecture, and regulatory signal transduction within the TME. These data establish immunotherapy as an efficient but limitedly applicable treatment strategy for EC, and drive the development of methods to regulate and integrate the microenvironment to expand immune responses beyond the traditional sensitive subtypes.

ICIs have reshaped the treatment landscape for EC, but their clinical impact is fundamentally limited by the tumor's biological characteristics [37]. The durable benefits are mainly confined to a subset of tumors with biological immunogenicity, while the majority of EC exhibit intrinsic or adaptive resistance. This significant heterogeneity can be best explained through molecular stratification analysis (rather than a simple treatment approach) (Fig. 3) [38].

Tumors with extremely high mutational burden (especially POLE-mutated epithelial cells) show abnormal sensitivity to PD-1/PD-L1 blockade therapy, with an ORR often exceeding 50%-60%. Tumors with MMR-d and MSI-H constitute a larger and clinically operational subgroup. In advanced or relapsed diseases, the objective response rate of multiple trials is approximately 30%-45% [39,40]. In contrast, the two main subtypes with low copy numbers (NSMP) and high copy numbers (TP53 mutations), mainly belonging to diseases with normal mismatch repair function/microsatellite stable type, have limited benefits from ICIs, with their response rates typically being lower than 10%-15%, even when combined treatments are used [41,42].

These reaction gradients reveal a fundamental principle: in solid EC, the efficacy of immunotherapy is influenced by the subtype-specific immune-limiting factors embedded within the TME, rather than the checkpoint targets themselves [43]. Therefore, clarifying the role of immunotherapy requires a shift from the checkpoint-centered paradigm to a restriction-centered framework. In this framework, both responsiveness and resistance need to be interpreted from perspectives such as antigenicity, immune initiation, transport, and inhibition. Under this framework, immunotherapy is not a universal solution but rather a highly effective component that depends on specific circumstances. Its clinical relevance can only be achieved through biological targeting - in many cases, active activation is also required [44].

#### 2.2.1 Immunotherapy is effective in EC only under defined biological conditions

ICB has the most significant clinical benefits in EC, which is observed in tumors with DNA repair defects or hypermutated characteristics. These tumors produce a large number of neoantigens and support pre-existing anti-tumor immune responses. POLE-mutant ECs, defined by pathogenic mutations in the exonuclease domain of DNA polymerase epsilon, most commonly P286R and V411L, exhibit extraordinarily high tumor mutational burden and dense infiltration by cytotoxic T lymphocytes [45]. Although rare, these tumors are among the most immunotherapy-sensitive solid malignancies described to date, with ORRs frequently exceeding 50-60% and responses that are often deep and durable [46].

MMRd or MSI-H ECs represent a larger proportion of patients and constitute the most clinically actionable immunotherapy-responsive subgroup [47]. Across multiple trials of PD-1 blockade in advanced or recurrent disease, MSI-H/MMR-d ECs consistently achieve ORRs in the range of 30-45%. In the KEYNOTE-158 trial, pembrolizumab monotherapy produced an ORR of approximately 48% in previously treated MSI-H/MMRd EC, with durable disease control. Similarly, the GARNET trial reported ORRs of ∼43-45% with dostarlimab in MSI-H/dMMR disease, outcomes that markedly exceeded those observed in mismatch repair-proficient cohorts [35,48]. These data have firmly established MSI-H/MMR-d status as a predictive biomarker for ICI benefit and underpin current regulatory approvals. By contrast, the majority of ECs, including NSMP and TP53-mutant tumors, exhibit limited responsiveness to ICB [49]. These subtypes typically exhibit a lower level of neoantigen load, sparse spontaneous immune infiltration, and a dominant network of immunosuppressive signals [50]. Clinical studies have shown that even when combined with ICIs, chemotherapy, anti-angiogenic drugs, or targeted therapies, the ORR for these populations is typically still below 10%-15%. These results indicate that relying solely on immune checkpoint inhibition is insufficient to break through the immune barrier of immunologically cold EC.

Based on these observations, it can be confirmed that high mutation load and basal immune activation are necessary conditions for the effectiveness of immunotherapy in EC, but they are not sufficient conditions. Even in the responsive subtypes, the heterogeneity of antigen presentation and immune regulation still affects the clinical outcome; while in the non-responsive subtypes, structured immune suppression significantly limits the therapeutic activity.

#### 2.2.2 Targeting immune regulatory axes: focusing on STAT3 and innate immune signaling

ICIs have limited efficacy in non-microsatellite instability high EC, which not only reflects the lack of immune effector cells, but also stems from the dominant role of the organized immunosuppressive network in the tumor microenvironment. Reduced infiltration of CD8^+^ T cells, expansion of regulatory T cells (Tregs) and myeloid-derived suppressor cells (MDSCs), impaired maturation of dendritic cells, and dysfunction of antigen presentation all contribute to the immunosuppressive state, which results in a poor response to treatments that merely release immune checkpoints.

Among the signaling pathways that regulate this inhibitory mechanism, the STAT3 axis has become the core integrator of immune, endocrine and metabolic signals in EC. The continuously activated STAT3 promotes the differentiation of Tregs, inhibits the function of dendritic cells (DC), and supports the survival of tumor cells, thereby coupling immune escape with proliferation and metabolic advantages. At the same time, the activation defect of the innate immune sensing pathways (especially the cGAS-STING protein axis) limits the signaling of type I interferons and effective immune initiation, and this phenomenon is particularly evident in tumors with low and high copy numbers. These research results indicate that to extend the benefits of immunotherapy for patients with EC, the key lies in the upstream reconfiguration of the immune ecosystem rather than simply strengthening ICB. Biomaterial delivery technology provides a mechanistic solution to this problem. By achieving local and continuous delivery of immune regulators such as STAT3 inhibitors and pain receptor agonists in the uterine or tumor microenvironment, the biomaterial platform can not only enhance the immune activation effect but also minimize the side effects limited by systemic toxicity caused by traditional methods [51].

In this context, the immunotherapy for EC has shifted from a single checkpoint-based intervention approach to being part of microenvironment-oriented immune reprogramming. Such strategies are particularly promising in transforming immunologically 'cold' EC subtypes into treatable disease states, thereby expanding the clinical application scope of immunotherapy beyond tumors with MSI-H and polarity protein deficiency (POLE) mutations.

### 2.3 Endocrine therapy beyond hormone suppression: integration with immune and metabolic control

Endocrine therapy remains the primary treatment approach for hormone-sensitive EC, especially for tumors with low copy number (NSMP), which are characterized by estrogen dependence and relatively stable genomes [33,34]. Progesterone and selective estrogen receptor modulators can induce tumor differentiation and inhibit their growth, providing a theoretical basis for fertility preservation treatment for patients who have undergone strict screening [52,53]. However, the clinical benefits are often short-lived. A significant proportion of cases develop acquired resistance, which not only limits the durability of the treatment but also highlights the necessity of adopting a combined treatment strategy based on biological principles.

A dominant mechanism underlying endocrine resistance in EC is compensatory activation of the PI3K-AKT-mTOR signaling axis, one of the most frequently altered pathways in this disease [54]. Activation of this pathway attenuates hormone sensitivity while simultaneously promoting tumor cell proliferation, metabolic reprogramming and immune evasion. Beyond tumor-intrinsic effects, PI3K-AKT-mTOR signaling reshapes the tumor microenvironment by enhancing glycolytic flux, suppressing antigen presentation and fostering the accumulation of regulatory immune cell populations. These interconnected effects provide a strong mechanistic rationale for integrating endocrine therapy with targeted inhibition of PI3K-AKT-mTOR signaling to restore hormonal responsiveness and recondition the immune-metabolic milieu [55,56].

Preclinical studies and early-phase clinical investigations support this concept. mTOR inhibitors such as everolimus and temsirolimus have been evaluated in combination with hormonal therapy in advanced or recurrent EC, based on the hypothesis that downstream pathway blockade can resensitize tumors to progestins. Although randomized evidence remains limited, available data indicate that PI3K-AKT-mTOR inhibition can prolong progression-free survival in selected settings. However, when these drugs are used for a long period of time, their significant systemic toxicity offsets the aforementioned benefits. Combined treatment strategies targeting other pathways such as the PI3K-AKT-mTOR pathway and the MAPK-ERK signaling pathway are also being actively studied, which further highlights the core role of pathway-driven drug resistance [57].

These limitations highlight a crucial transformation bottleneck: Although the biological basis for the integration of the endocrine pathway is convincing, systemic administration limits its feasibility, especially for young patients, where long-term treatment tolerance and reproductive function are crucial. In this context, the local delivery strategy enabled by biomaterials provides a practical solution for achieving endocrine-targeted combined therapy while reducing systemic exposure. By implanting injectable hydrogels or biomaterial platforms into the uterine cavity, it is possible to simultaneously release progesterone and PI3K-AKT-mTOR inhibitors at the junction between the tumor and the endometrium, thereby maintaining therapeutic concentrations locally while minimizing off-target effects.

It is worth noting that the functions of this type of biomaterial platform are not limited to spatial positioning. By virtue of its continuous and programmed release kinetics, these systems can precisely control the drug ratio and coordinate the temporal relationship between endocrine intervention and pathway blockade. This regulatory mechanism can simultaneously continuously inhibit hormone-driven growth while selectively blocking drug-resistant pathways - this balance is difficult to achieve with systemic medication. Therefore, this strategy is particularly suitable for drugs targeting metabolic or growth signaling pathways, as these drugs are often difficult to be used for long-term treatment to preserve fertility due to their toxic characteristics. In addition to directly controlling tumors, integrating endocrine-metabolic regulation also has broader immunological significance. Tumor differentiation induced by endocrine therapy, when combined with the weakened metabolic program driven by PI3K-AKT-mTOR, can form a microenvironment more conducive to immune surveillance. In this context, the biomaterial serves as a coordinating interface, integrating endocrine regulation and metabolic normalization, and combining with the immune response to form a unified treatment framework.

The repositioning of endocrine therapy - from a single inhibitory treatment mode to an environment-setting regulator of tumor-immune-metabolic interactions-represents a transformation in the treatment concept for hormone-sensitive EC. This transformation is particularly important in fertility preservation treatment, as long-term systemic endocrine intervention is neither ideal nor sustainable. Through spatial limitations and time-programmed endocrine and targeted pathway inhibition, the biomaterial-assisted strategy provides a feasible, subtype-based treatment path for clinical practice, enhancing therapeutic synergy while reducing adverse reactions.

### 2.4 Metabolic targeting as a convergence point for immune and endocrine resistance

Metabolic dysregulation is a distinctive feature of EC, linking obesity, hyperinsulinemia, chronic inflammation, and steroid metabolism abnormalities to tumor progression [58,59]. Mechanistically, several metabolic nodes are closely associated with EC treatment resistance: aerobic glycolysis and lactic acid accumulation acidify the tumor microenvironment, impairing the function of cytotoxic T lymphocytes and dendritic cells; glucose utilization and anabolic signaling induced by PI3K-AKT-mTOR promote endocrine escape and sustained proliferation; fatty acid synthesis and β - oxidation provide the energy and flexibility needed for membrane construction in invasive diseases; local estrogen biosynthesis mediated by aromatase and associated lipid-steroid pathways promotes hormone-dependent growth. These alterations not only promote tumor expansion but also reshape the disease's immunological and endocrine environment, thus establishing metabolism as a biological link between immune resistance and endocrine insufficiency [60,61].

Targeting these pathways alone provides only limited benefits, partly because systemic metabolic inhibitors generally have narrow therapeutic windows and tumor cells can compensate through parallel nutrient utilization pathways. Biomaterial delivery technologies offer more selective intervention methods. In principle, nanotransporters, long-acting intrauterine devices, and injectable hydrogels can be used to deliver glycolytic signaling inhibitors (e.g., targeting hexokinase-2, LDHA, or lactate transporters such as MCT1/MCT4), energy stress pathway modulators (e.g., AMPK activators, glutamine utilization inhibitors), or compounds that mitigate lipid synthesis and SREBP/FASN-related programs. Although the evidence in endocrine cancers remains mainly pre-clinical, studies on gynecological tumor models and other solid tumors indicate that localized or prolonged delivery of these agents can reduce lactic acid burden, improve intratumoral pH balance, mitigate myeloid and T cell suppression, and restore tumor cell sensitivity to endocrine or immune therapies. Biomaterials are particularly promising in this context because they can maintain therapeutically relevant local concentrations while mitigating the systemic metabolic toxicity that limits the long-term systemic delivery of these agents.

The therapeutic benefits of treatments that target specific metabolic pathways alone are limited, partly due to systemic toxicity and metabolic redundancy. Biomedical delivery technology offers a new approach for selectively regulating tumor-related metabolism, effectively reducing immunosuppressive by-products and thereby reshaping a metabolic state conducive to immune activation [62]. The combination of metabolic regulation with immunotherapy or endocrine intervention can lower the threshold of resistance and improve therapeutic synergy. Since the associations between different EC subtypes and specific metabolic targets are diverse, a stratified prioritization strategy based on subtypes is required. Thanks to its modular design, this biomaterial platform allows for flexible adaptation of intervention methods: the metabolic intervention can be selectively integrated according to the biological characteristics of the tumor, rather than being applied uniformly in a standardized manner (Table 1).

**Table 1:** Mapping of EC molecular subtypes to biomaterial platforms and personalized therapeutic strategies. EC covers various molecular subtypes in biology, including pole mutant, MMR-D/MSI-H, CN-Low (NSMP) and CN-high (TP53-mutant), each with unique immune, endocrine and metabolic limitations that affect treatment sensitivity and resistance. The table integrates the specific biological characteristics of the subtype with a rational selection of biomaterial platforms and the corresponding load design, with the aim of harmonizing treatment modalities, delivery strategies and phenotypic limits. For immunogenic POLE and MSI-H mutant tumors, pH nanovectors or protease reagents with hydrogel reservoir can facilitate local immune initiation by PD-1/PD-L1 inhibitors and innate immunomodulators, thereby reducing systemic toxicity while enhancing endogenous antitumor response. In CN-low- hormonal diseases, continuous and compartmentalized endocrine regulation delivered by the uterine storage or scaffolding system minimizes systemic exposure while enhancing hormonal control and matrix-epithelium synchronization. For CN-high tumors with treatment resistance and normal pMMR/MSS diseases, modular, multi-stimulus-responsive platforms can combine the delivery of cytotoxic drugs, immunomodulators and metabolic agents, aiming to reshape the inhibitory microenvironment. In different subtypes, the main goals and candidate evaluation indicators range from improving objective response rate and progression-free survival in advanced treatment to maintaining uterine endometrial integrity and restoring reproductive function in fertility-aware care. This map demonstrates how biological-guided material design can achieve treatment integration, providing a framework for precise multimodal treatment of EC

EC Molecular Subtype	Biological Constraint	Biomaterial Platform	Therapeutic Payload(s)	Primary Goal/Readout
POLE-mutant	High immunogenicity; high neoantigen load	**pH- or enzyme-responsive nanocarrier**	PD-1/PD-L1 inhibitors; STING agonists	Enhanced local immune priming; high ORR
MSI-H/dMMR	Immune-responsive but TME suppression	**Protease-degradable hydrogel**	ICI ± TME modulators (STAT3 inhibitors)	Durable responses; reduced systemic irAE
CN-low (NSMP)	Endocrine driven; immune cold	**Sustained-release intrauterine depot**	Progestins ± PI3K-AKT-mTOR inhibitors	Enhanced endocrine response, local control
CN-high (TP53-mut)	Aggressive biology, resistant to monotherapy	**Gradient multifunctional scaffold**	Chemo ± immuno ± metabolic modulators	Bridge tumour suppression + microenvironment reconditioning
pMMR/MSS	Immune resistant	**Multi-stimuli responsive nanoparticles**	Redox/pH triggered immuno + metabolic agents	Convert cold to hot TME

From the perspective of translational medicine, the most promising metabolic strategies involve integration into existing subtyp-specific treatment regimens rather than an isolated intervention. For hormone-sensitive diseases with low copy number alterations, administration of progestogens via biomaterials in combination with PI3K-AKT-mTOR modulators or glycolysis may help maintain endocrine sensitivity while limiting systemic adverse effects. In altered pMMR or TP53 immunocompromised tumors, localized release of lactate reducing or immunopermissive metabolic modulators may improve antigen presentation and t-cell recruitment prior to or during control point blocking. Similarly, in fertility preservation scenarios requiring balanced and lasting tumor control with preservation of endometrial function, peritoral or intrauterine platforms delivering sequential metabolic normalizers followed by immunotherapy or regenerative signaling may be of particular interest. From this point of view, biomaterial-guided metabolic reprogramming does not represent an accessory complement, but a practical layer to reduce resistance thresholds in endocrine and immunotherapeutic paradigms.

## 3 From tumor control to endometrial regeneration: biomaterial-driven integrative design

Effective management of EC, particularly in patients seeking fertility preservation, requires a shift from a tumor centered intervention strategy to a comprehensive treatment regimen, while balancing tumor control with the integrity of uterine function. Although multimodal treatment can inhibit tumor growth, its long-term effectiveness in patients of childbearing age ultimately depends on preservation or reconstruction of the endometrium with reproductive function. The unique strength of this biomaterials platform lies in its positioning in this interdisciplinary field, allowing the integration of antitumor efficiency and endometrial regeneration into a unified therapeutic framework.

### 3.1 Biological basis of endometrial injury and failed regeneration

**Fig. 4:**
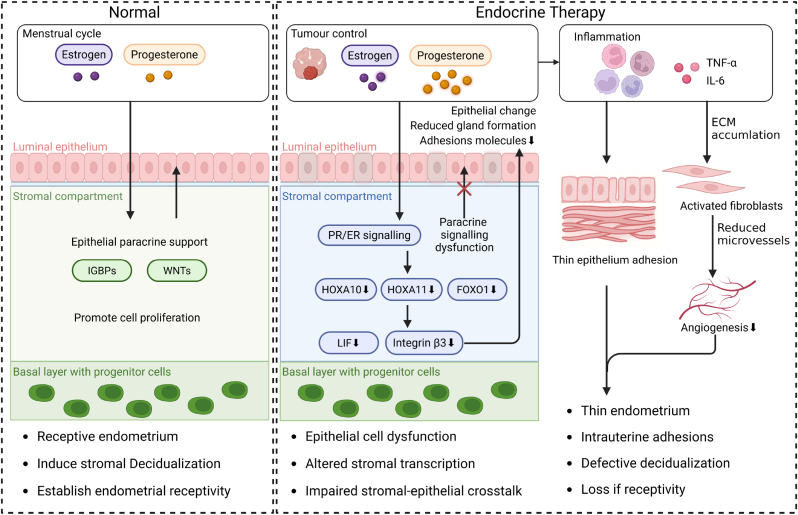
**Endocrine therapy driven mechanisms of endometrial injury and failed regeneration.** The endometrium is a dynamic tissue that undergoes periodic regeneration, which relies on a complete basal layer, coordinated intercellular communication between the stroma and epithelial cells, and strictly regulated ovarian steroid signaling. Surgical intervention and long-term endocrine therapy disrupt these regenerative processes through convergent structural, transcriptional and inflammatory mechanisms. Continuous exposure to progesterone can inhibit tumor growth and simultaneously alter the PR-dependent transcription in stromal cells, leading to dysregulation of paracrine signals between epithelial cells. The core molecular consequence is the downregulation of homeobox transcription factors HOXA10 and HOXA11, which are necessary for stromal decidualization, epithelial differentiation and adhesion molecule expression related to implantation. The absence of HOXA10-dependent signaling releases the synchrony between stroma and epithelium, impairs glandular maturation and reduces endometrial receptivity. At the same time, endocrine disorders interact with tumor and metabolic-related inflammation to promote fibroblast activation, ECM deposition and microvascular sparseness, thereby leading to tissue remodeling towards fibrosis rather than regeneration. These processes ultimately result in thinning of the endometrium, intrauterine adhesions and decidualization defects, constituting failure of regeneration, which is a common major therapeutic endpoint in the treatment and management of EC with reproductive awareness.

Effective management of EC, particularly in patients seeking fertility preservation, requires a shift from a tumor centered intervention strategy to a comprehensive treatment regimen, while balancing tumor control with the integrity of uterine function. Although multimodal treatment can inhibit tumor growth, its long-term effectiveness in patients of reproductive age ultimately depends on preservation or reconstruction of the endometrium is a highly dynamic mucosal tissue that undergoes periodic proliferation, differentiation and drop under the strict regulation of ovarian steroid hormones. Its intrinsic regenerative capacity depends on the integrity of the basal layer, accurate communication between the stromal and epithelial cells, and coordination of the transduction of endocrine signals. Together, these factors ensure that tissues renew themselves after menstruation or pregnancy, restore the ability to conceive and rebuild function. However, this precisely regulated regeneration process is extremely vulnerable to iatrogenic and disease-related damage, which can lead to disruption of tissue structure, Paracrine communication and gene regulation networks and ultimately compromise reproductive potential (Fig. 4) [63].

Structurally, endometry regeneration relies on preservation of the basal layer, which contains resident populations of stem cells essential for periodic epithelial renewal, matrix support and vascular remodeling. Surgical procedures commonly used in the diagnosis and treatment of EC, such as diagnostic curettage and tumorectomy to preserve fertility, can directly damage the area. Even with limited macroscopic damage, destruction of the basal layer can create structural bottlenecks that limit further repair and lead to persistent defects in epithelial regeneration, stromal tissue and angiogenesis. These lesions often have long-term effects on endometrial function and are not commensurate with apparent tissue loss [31].

In addition to obvious structural damage, endocrine therapy, especially prolonged or high dose progesterone exposure is a more subtle but equally important factor contributing to endometrial dysfunction. Although progestogens play a central role in fertility preservation treatment for hormone-sensitive EC by inducing tumor differentiation and growth arrest, continuous progestogen signaling can profoundly reshape the cellular and transcriptional landscape of the endometrium. At the cellular level, normal regeneration requires close coordination between epithelial cell proliferation and stromal decidualization-both of which are basically regulated by progestogen receptors (PR) by progestogens. The long-term activation of this axis disrupts the balance of hormone signals and paracrine mediators necessary for effective stromal-epithelial communication, thereby leading to decidualization impairment and receptor dysfunction. The key molecular node linking endocrine disorders to regenerative disorders is the homeobox transcription factor HOXA10 and its closely related paralog HOXA11 [64,65]. These genes are induced to be expressed during the secretory phase under the combined action of estrogen and progesterone, and play a crucial role in establishing endometrial receptivity, matrix differentiation, and implantation ability [66,67]. HOXA10 regulates the transcriptional program that determines the responsiveness of the stroma to progesterone, epithelial differentiation, cell adhesion, and immune regulation. Persistent or dysregulated endocrine regulation is associated with the downregulation or epigenetic silencing of HOXA10 and HOXA11, leading to disruption of epithelial-stromal synchrony, impaired glandular development, and reduced expression of adhesion molecules required for embryo implantation [68,69].

The experimental model revealed the central role of this pathway, the loss of the HOXA10 gene disrupts the responsiveness of the stromal cells to progesterone, resulting in mucosal defects, abnormal transduction of the Paracrine signal and implantation failure. In human pathology, abnormal HOXA10 expression is commonly found in diseases of impaired endometrial function, including EC, endometriosis, and recurrent implantation failure, which are associated with reduced receptor sensitivity and decreased reproductive success. In addition to transcriptional regulation, new evidence suggests that HOXA10 also restricts epithelial plasticity by inhibiting the mesenchymal gene program in the luminal epithelium. The downregulation of HOXA10 allows for partial epithelial-mesenchymal transition (pEMT) like states, which may be physiologically necessary for implantation, but when dysregulated, it disrupts epithelial integrity and hinders regenerative remodeling [70]. In this study, the HOXA10 and HOXA11 genes are mainly addressed in relation to the functional axis of the endometrium, serving as indicators of receptivity, stromale-epithelial coordination and regenerative capacity. Therefore, this article mainly utilizes these mechanisms to explain the treatment of related endometrial functional disorders and the repair of damaged pathological processes, rather than suggesting that they have a direct tumor-promoting effect in EC.

Inflammation is another important driving factor for the damage and regeneration of the endometrium. However, this mechanism is often overlooked. Tumor-associated inflammation, treatment-induced immune activation, and chronic low-grade inflammatory states associated with obesity and metabolic dysfunction jointly disrupt the local microenvironment of cytokines and growth factors. Elevated levels of pro-inflammatory cytokines and matrix remodeling enzymes (including matrix metalloproteinases) promote fibroblast activation, excessive extracellular matrix deposition, and microvascular sparsification. These factors collectively lead to fibrosis rather than regeneration. It is worth noting that endocrine disorders and inflammation are not independent processes: hormonal disorders amplify inflammatory signal transduction, further disrupting the matrix-epithelial communication and inhibiting angiogenic responses [71,72].

When structural damage, endocrine imbalance and inflammation interweave with each other, even if the tumor is effectively controlled, the regeneration of the endometrium may still fail. Clinically, the consequences are not limited to reduced fertility; they can also lead to the permanent loss of reproductive potential, manifested as thinning of the endometrium, uterine cavity adhesions, and defects in decidualization. These lesions will seriously impede embryo implantation and pregnancy maintenance. Therefore, in the treatment of EC focusing on reproductive function, the failure of endometrial regeneration should not be regarded as a secondary or accompanying outcome, but should be regarded as a common primary treatment endpoint closely related to the success of tumor treatment, and must be resolved through reasonable and comprehensive treatment plans [72].

### 3.2 Unique value of biomaterials in post-treatment repair and regeneration

**Fig. 5:**
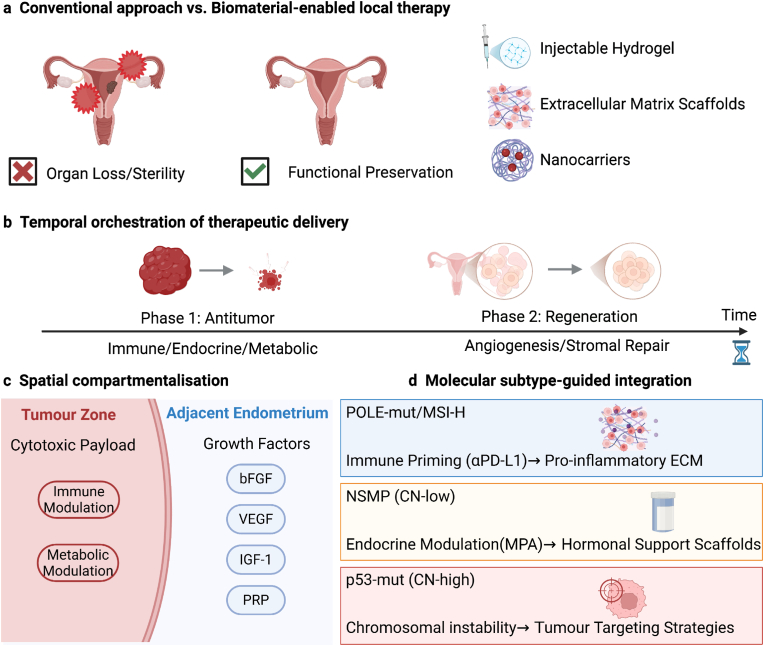
**Biological materials support post-treatment endometrial repair by providing structural guidance, local bioactive signal transduction, and a microenvironment conducive to regeneration, thereby linking fertility preservation with oncological management.** The platform based on biomaterials has achieved a transformation from tumor-centered treatment to a comprehensive strategy, which can simultaneously achieve tumor control and the preservation or restoration of endometrial function. Although traditional systemic treatments aim to completely eliminate tumors as their primary goal, they often damage the integrity of the endometrium and reproductive functions. In contrast, local biological material systems (such as injectable hydrogels, nanocarriers, and ECM mimetic scaffolds) can achieve precise temporal and spatial control of therapeutic delivery in the uterine environment. These platforms can coordinate the sequential or regional release of anti-tumor drugs (including immunomodulatory, endocrine, and metabolic therapies), and then release regenerative signals that support matrix repair, angiogenesis, and epithelial renewal. Importantly, biomaterial integration technology enables the treatment design to be matched with molecular subtypes, disease stages, and regenerative needs, rather than being uniformly applied. By integrating regenerative intentions from the very beginning of the treatment design, biomaterials serve as a bridging infrastructure, connecting precision oncology with reproductive medicine, and redefining fertility preservation as a primary therapeutic goal rather than a consideration after treatment.

The traditional methods for repairing the endometrium mainly rely on systemic hormone supplementation or empirical adjuvant therapy, but the therapeutic effects vary. In contrast, the strategy based on biomaterials can more precisely simulate the natural regenerative microenvironment of the endometrium by introducing spatial, mechanical and biochemical regulations (Fig. 5).

Simulated injectable Hydrogel and extracellular matrix (ECM) scaffolding provides temporary structural support in the uterine cavity, promoting cell adhesion, migration and angiogenesis. By simulating the viscoelastic properties of natural ecm with biochemical signals, these materials create a microenvironment conducive to tissue repair rather than passive wound filling. Importantly, its degradability can be adjusted to match the endometrial regeneration time scale, thereby minimizing the long-term response to foreign bodies [72,73]. Biomaterials not only have a structural support function, but can also serve as a multifunctional support for biologically active substances. Local administration of stem or progenitor cells, angiogenic growth factors and immunomodulatory signals can not only contribute directly to the regeneration process, but also avoid systemic exposure [74]. For example, mesenchymal stem cells delivered by scaffolds of biomaterials can have paracrine effects that promote angiogenesis, inhibit fibrosis and regulate inflammation - functions that are particularly important in the uterine environment after treatment.

The evidence supporting this regenerative concept comes mainly from preclinical models of endometrial lesions. Representative studies demonstrate that local administration of stem/progenitor cells or bioactive molecules, using injectable hydrogels, biomimetic decellularized extracellular matrices and collagen or polysaccharide-based biomaterials, can improve endometrial thickness, promote gland formation, reduce fibrosis and improve angiogenesis. At the same time, biomaterial-assisted administration of growth factors or secretomes rich in extracellular vesicles has been shown to improve endometrial receptivity characteristics in damaged endometrial tissues. Although these results have not yet established tumor safety in the specific context of endometrial carcinoma, they provide a proof of concept demonstrating the biological feasibility of structural and functional repair of the endometrial guided by biomaterials. However, important gaps remain in this area, including the need to establish subtype-specific disease models, to obtain long-term data on reproductive outcomes, and to define criteria for determining when regenerative interventions can be implemented without compromising tumor control.

The key is that the role of biological material in this situation should not be seen as a remedy for post-treatment damage. Instead, their regenerative ability redefines the treatment goal. Endometrial repair is not an auxiliary measure after tumor eradication, but must be anticipated from the very beginning and incorporated as an integral part of the treatment design. This shift in perspective distinguishes the strategy supported by biological materials from traditional reproductive preservation methods, and elevates regeneration to a common primary treatment goal [75].

### 3.3 Design paradigms for integrated therapy and regeneration

To achieve the true integration of tumor control and endometrial regeneration, the mere combination of anti-cancer drugs and regenerative drugs is not sufficient; it requires the rational design principles for coordinating therapeutic effects in terms of time, space and biological context. Biomaterials provide a platform for achieving this coordination, through which treatment plans can be systematically implemented, converting conceptual integration into executable treatment plans [76].

The time coordination mechanism is the core pillar of integrated design. The time-resolved delivery system enables the treatment priority to evolve dynamically in accordance with the disease, allowing anti-tumor activity to dominate the early treatment stage, while regenerative signals are introduced after the tumor burden is under control. Sequential release architectures minimize antagonistic effects between cytotoxic or immunomodulatory interventions and tissue repair processes, thus promoting a controlled transition from tumor suppression to endometrial repair. This time interval is particularly important in the context of reproductive health awareness, as premature activation of regeneration pathways can weaken tumor control, while persistent anti-tumor stress can irreversibly compromise endometrial repair.

The spatial zoning technique further improves the accuracy of the treatment by exploiting the anatomy of the uterus and the heterogeneity of the microenvironment. This biomaterial platform makes it possible to physically isolate tumor-targeting drugs from components that promote regeneration, thus enabling local immune or metabolic regulation at the tumor site while supporting endometrial repair of adjacent tissues. In this context, growth factors and biological activity signals such as alkaline fibroblast growth factor (bFGF), vascular endothelial growth factor (VEGF), insulin-like growth factor 1 (IGF-1) or platelet rich plasma (PRP) can be released in areas that are not significantly affected by high antitumor exposure [[77], [78], [79]]. This spatial partitioning is particularly important in EC because tumor infiltration is usually focal rather than diffuse, which allows the regenerative microenvironment to be preserved or reconstructed without affecting the oncological safety.

The molecular subtype matching provides a biological basis for the integration of truly individualized treatment in EC. Each subtype imposes unique restrictions on immune function, endocrine dependence, and regenerative capacity, not only determining the required treatment approach but also its timing and implementation method. Tumors with strong intrinsic immunogenicity (such as POLE mutant and MSI-H diseases) are most suitable for early immune activation. At this time, rapid release of anti-tumor immunity can achieve durable tumor control. In such cases, regenerative support should be delayed as much as possible, and introduced only after the immune-mediated clearance is established, thereby minimizing interference between immune activation and tissue repair. In contrast, hormone-driven low-copy number tumors are characterized by persistent endocrine dependence and relative genomic stability, and require long-term hormone regulation and ECM-guided tissue repair combined with the maintenance of the integrity and function of the endometrium. High-copy number tumors are marked by chromosomal instability and invasive biological characteristics, with additional restrictions, and usually require an intensified tumor-directed treatment strategy before considering the regenerative factors for safety.

The modular characteristics of the biomaterial system enable this subtype-specific customization to be achieved in practice. By allowing flexible composition, programmable release kinetics, and spatial compartmentalization, the biomaterial platform can adapt to the unique biological needs of each molecular subtype, ensuring that the integration process is not uniformly imposed but is based on biological information, time-sequenced, and context-dependent.

These design principles jointly elucidate how biomaterials promote the conceptual shift from combined treatment to coordinated treatment, this shift is achieved simultaneously within a single framework that focuses on reproductive function, achieving tumor control and endometrial regeneration. By directly embedding time, space, and biological logic into the treatment delivery process, the biomaterial-enabled strategy provides a practical and clinically consistent path for achieving the coordination of oncological efficacy and the recovery of uterine structure and function.

Despite this perspective, EC regeneration strategies should be interpreted with caution, particularly in fertility preservation treatments where tumor safety and tissue repair coexist in a delicate balance. Endometry repair after treatment is not inherently benign: premature or poorly targeted administration of regenerative growth factors, matrix remodeling signals, or pro-angiogenic agents may facilitate survival of residual tumor cells, increase stromal permissiveness, or cause disease recurrence. Other uncertainties include the interpatient variability of regenerative responses, the incomplete understanding of how subtype-specific tumor biology interacts with post-treatment repair programs, and the lack of long-term data on the association between biomaterial assisted regeneration and reproductive outcomes in conjunction with tumor persistence. These factors highlight the need for spatially limited, time-staggered biomaterials design, introducing regenerative signals only after adequate tumor control has been achieved, and monitoring in a setting that treats tumor safety and endometrial function restoration as inseparable therapeutic objectives.

## 4 Clinical translation: regulatory, manufacturing and clinical pathways for making subtype-guided integration actionable

The clinical translation of subtype-oriented strategies enabled by biomaterials in EC is successful not only depending on the technical complexity. Although preclinical studies have confirmed its significant biological basis, for effective implementation, it is still necessary to ensure the coordination among the therapeutic logic, clinical trial design, and the medical service system. Understanding the strengths and weaknesses of existing methods is crucial for transforming the integrative concept into clinically meaningful treatment outcomes.

### 4.1 Lessons from existing clinical trials: why biologically sound strategies underperform

In the field of EC, clinical studies that combine immunotherapy with endocrine or targeted drugs have demonstrated that multimodal treatment can achieve better therapeutic effects than single therapy. Trials that combine immune checkpoint inhibitors with hormone therapy or PI3K-AKT-mTOR pathway inhibitors have shown that this regimen exhibits stronger therapeutic activity in specific patient subgroups (especially hormone-sensitive or MMR-d disease patients) [80]. However, these benefits are often limited, inconsistent, or limited to specific groups as defined narrowly.

For a long time, the systemic administration method has had an inherent limitation: it not only amplifies the off-target risk, restricts dose escalation, but also masks subtype-specific effects, especially for immunomodulators with narrow therapeutic windows. In contrast, local administration has been explored for a long time in the field of gynecological tumors, but modern clinical trial designs have not been fully applied. Its advantage lies in achieving targeted therapeutic effects within the uterus while reducing systemic toxicity. This biopolymer-supported local administration platform provides a mechanistic basis for re-examining this approach, and its continuous exposure and synergistic release characteristics are beyond the reach of traditional systemic administration schemes.

It is worth noting that the failure of many clinical strategies is not due to the deficiency of their biological basis, but rather due to the lack of systematic consideration during implementation. Immunotherapy is often introduced without synchronously regulating immune suppression pathways, endocrine therapy is not accompanied by correction of metabolism or microenvironment, and considerations related to regenerative medicine are often only included after the treatment. Therefore, these trials often validate the theoretical logic of combination therapy, but there are flaws in the implementation level, and the clinical results obtained are biologically reasonable, but fail to achieve the expected clinical effects.

### 4.2 Fertility-preserving populations: redefining trial design and success metrics

In clinical studies of EC, the patient population seeking fertility preservation is unique and underrepresented. Incorporating these studies requires breaking away from the traditional clinical trial framework - which often focuses more on the maximum tumor response rather than the long-term functional outcome. In this context, patient choice becomes a key factor in determining the safety and effectiveness of treatment. Indicators such as molecular subtype, tumor grade, depth of infiltration and initial endometrial reserve should be included in the inclusion criteria to ensure that the biological characteristics of patients are consistent with the adequacy of fertility oriented treatment strategies.

It is also essential to redefine the parameters of treatment. Traditional indicators such as objective remission rate or progression-free survival reflect only a part of the clinical benefit of this population. Indicators such as measurement of endometrial thickness, pregnancy markers, menstrual cycle recovery and pregnancy success are essential additional parameters. Biomaterial-assisted methods, by clearly targeting regeneration and tumor control, require the use of a composite outcome framework that reflects both oncological and reproductive success [81].

Long-term safety and follow-up are particularly crucial. The intervention measures for maintaining pregnancy not only need to prove their lasting tumor suppression effect, but also need to ensure that they do not cause delayed adverse effects on uterine function, pregnancy outcomes, and the health of the offspring. These requirements highlight the necessity of conducting long-term monitoring and interdisciplinary collaboration among oncologists, reproductive experts, and materials scientists throughout the entire clinical life cycle.

### 4.3 Translational barriers: a systems challenge rather than a materials problem

Although the field of biomaterial design is experiencing rapid innovation, its clinical application is still constrained by systemic obstacles. Producing complex multi-component platforms (especially those containing cells or bioactive molecules) under Good Manufacturing Practice (GMP) conditions poses challenges. However, with the advancements in scalable synthesis, standardization, and regulatory science, these obstacles are becoming increasingly surmountable.

Repeatability is another key challenge. If variability in material composition, release kinetics and biological response is not strictly controlled, this will affect clinical reliability. The establishment of standardized characterization indicators and quality criteria is therefore essential to ensure consistency between batches and between clinical trial centers. Perhaps the most underestimated obstacle is the coordination of clinical routes. Biomaterial-assisted integrative therapies are often not fully integrated into existing treatment options or reimbursement systems. Its successful implementation requires multidisciplinary collaboration between surgeons, physicians and reproductive oncologists, as well as early communication with regulatory agencies to clarify the appropriate route of approval. In the absence of such coordination, even a well-designed treatment can remain in the experimental stage. These considerations show that the main challenges of clinical transformation are at the organizational and procedural level rather than at the technical level. The resolution of these problems is essential to achieve routine clinical integration based on subtype guidance and biological material.

To overcome these barriers to translation, the following practical strategies can be adopted. Firstly, by early engagement with regulatory agencies, such as the US Food and Drug Administration (FDA)/European Medicines Agency (EMA), through breakthrough device/drug combination identification mechanisms, the regulatory requirements for combined biomaterial-drug products can be clarified, and the clinical endpoints can be aligned with regulatory expectations. Secondly, forming an alliance of material scientists, clinical physicians, and biostatisticians to develop standardized preclinical testing methods (such as organoids + in vitro uterine models) will enhance reproducibility and translation fidelity. Third, adaptive trial designs (e.g., basket or platform studies with biomaterial-assisted arms) can efficiently evaluate subtype-guided, locally delivered regimens while capturing long-term fertility and immunologic outcomes. In conclusion, these considerations indicate that the main challenges in clinical translation go beyond the material properties themselves and extend to regulatory classification, large-scale production, quality control, and coordination of clinical pathways. Addressing these interdependent constraints is crucial for achieving the integrated application of subtype-guided biomaterials in routine clinical care.

### 4.4 Regulatory pathways and scalable manufacturing: from proof-of-concept to deployable products

The transfer route for biomaterial-assisted EC therapy is influenced not only by biological efficacy but also limited by regulatory classification. Most of the platforms discussed in this study are not likely to be regulated as single drugs; Rather, they are likely to be classified as combination products, for which the material matrix, drug load and, in some cases, regenerative or cellular components need to be evaluated as an integrated therapeutic system. This has important implications for preclinical trials, as regulatory authorities generally require not only evidence of antitumor activity, but also an assessment of local biocompatibility, degradation behaviour, reproducibility of release, uterine safety, reproductive toxicity and device/drug interaction characteristics. For indications of reproductive function, the thresholds of evidence may be high, as dimensions such as preservation of endometrial function, implantation capacity and subsequent pregnancy outcomes have been changed from optional test criteria to critical indicators of clinical safety.

Large-scale manufacturing presents other challenges. Biomaterial systems that demonstrate laboratory excellence often rely on preparation processes, material heterogeneity or multi-step loading procedures that are difficult to replicate on a clinical scale. Therefore, translational applications favour platforms with defined composition, batch to batch consistency, sterilization compatibility, manufacturable release kinetics, and stability under practical storage conditions. The complexity itself is not insurmountable, but each additional component, be it a second drug, a bioactive polymer modification or a regenerative factor, adds to the burden of quality control and process validation. Therefore, phased development strategies may be particularly useful in the EC field: relatively simple intrauterine prolonged release devices or injectable hydrogels with clearly measurable release characteristics may represent more immediate pathways to clinical application than highly complex multifunctional structures.

These regulatory and industrial realities should not only be seen as obstacles; They can also optimize translational design. Early alignment of biomaterial structures with quality principles through design, GMP compatible synthesis pathways and clinically viable administration protocols significantly improves the potential for further application. Concretely, this means giving priority to materials that are proven to be safe, establishing test frameworks linking physicochemical properties to biological performance, and designing early human studies around clinically interpretable evaluation criteria such as local tolerance, pharmacodynamic modulation, pathological responses and endometrial recovery. This strategy bridges the remaining gap between conceptual innovation and applicable therapies, while providing a more complete clinical maturation pathway for biomaterial-assisted treatments targeted at EC subtypes.

## 5 Future directions: from multimodal integration to intelligent and individualized therapy

**Fig. 6:**
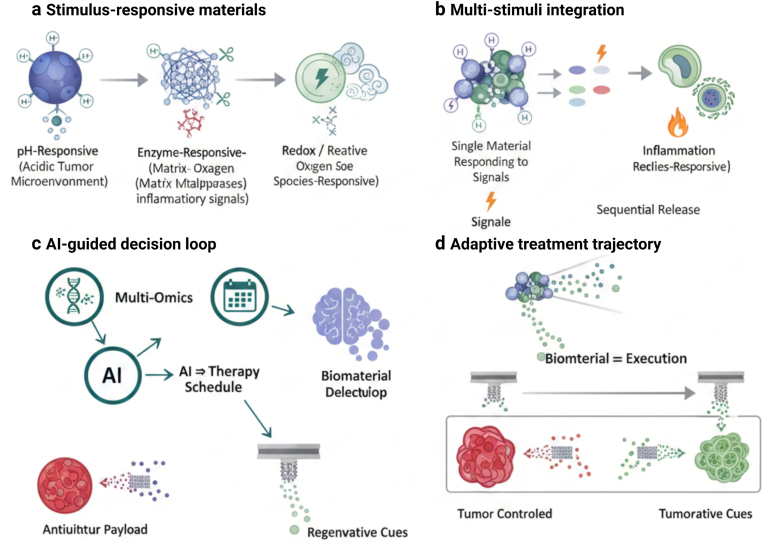
**The combination of intelligent biomaterials and AI-assisted stratification technology enables adaptive individualized treatment by matching drug delivery and release with the tumor's biological characteristics, treatment stage, and regenerative needs.** Stimuli-responsive (intelligent) biomaterials provide a platform for adaptive therapy by dynamically coupling the release of drugs with local microenvironmental signals. Endogenous triggering factors - including acidic pH, protease activity (such as matrix metalloproteinases), redox imbalance, and inflammatory signals, can be utilized to achieve the specific context activation of nanocarriers and hydrogels within tumors or injured tissues. The multi-stimulus response system further optimizes spatial and temporal control by integrating sensitivity to multiple biochemical signals, thereby achieving gradient or sequential release patterns as the disease state or regeneration stage evolves. Exogenous stimuli (such as light or ultrasound) provide additional external controllable precision within the accessible anatomical structures. It is important to note that intelligent biomaterials offer a physical interface through which ai-guided treatment decisions can be implemented: predictive models can infer tumor activity, immune status, or regeneration preparedness, and are connected to pre-programmed materials to respond accordingly. In this way, intelligent biomaterials act as transformation actuators, transforming digital decisions into localized and biologically appropriate therapeutic actions.

The next phase of progress in EC therapy will not be defined by the addition of new treatment modalities, but by the emergence of intelligent systems capable of dynamically aligning therapeutic delivery with tumor biology and patient-specific needs. As biomaterial-enabled multimodal strategies mature, their evolution towards intelligent and individualized frameworks will be essential to fully realize precision and fertility-conscious care (Fig. 6).

### 5.1 AI-driven subtype stratification and therapeutic decision-making

Molecular subtype classification has become the core of precision oncology for EC, but its clinical application remains largely static. The current classification schemes only capture the tumor biological characteristics at a single time point, and often fail to reflect the temporal evolution under treatment pressure. Artificial intelligence (AI) offers the opportunity to transform the stratification of subtypes of descriptive tools into predictive and adaptive decision engines [82,83].

By integrating multiomic data, imaging signatures, histopathologies and clinical variables, ai based models can identify potential biological models that accurately define subtype boundaries and predict treatment sensitivity. Such approaches are particularly important to guide multimodal integration, where the relative contributions of immunotherapy, endocrine regulation and metabolic targeting need to be dynamically optimized. It is important to note that the ai framework not only guides the choice of drugs, but also optimizes administration strategies and identifies patients most likely to benefit from local biomaterial-assisted intervention rather than systemic treatment.

In the area of fertility preservation, AI assisted decision support systems can further integrate reproductive parameters to achieve an individualized balance between tumor risk and reproductive potential.

### 5.2 Intelligent biomaterials: responsive platforms for adaptive therapy

Traditional biomaterials usually have fixed physical and chemical properties and pre-set release curves. Although such designs can achieve basic controlled release, their inherent limitations make them difficult to adapt to the spatial and temporal heterogeneity of the tumor microenvironment and the dynamic changes of the treated tissues. In solid EC where tumor biology, immune activity, and regeneration processes evolve over time, these rigid constraints limit the precision of treatment. These limitations have driven the emergence of intelligent stimulus-responsive biomaterials, which can undergo structural or functional changes in response to specific endogenous or exogenous signals, thereby achieving on-demand and context-specific therapeutic effects [84].

One type of major intelligent system utilizes the endogenous biochemical characteristics of the tumor microenvironment. Among them, pH changes are one of the most extensively studied triggering factors. Enhanced aerobic glycolysis and impaired perfusion cause the tumor area to be slightly acidic, with the extracellular pH typically ranging from 6.5 to 6.9, while the pH in normal tissues is close to neutral. pH-responsive nanocarriers and hydrogels are usually constructed from polymers with ionizable functional groups (such as carboxyl or amino groups), which undergo proton-dependent conformational changes or bond breaks under acidic conditions. These physical and chemical transformations alter the solubility, swelling behavior, or network integrity of the polymers, thereby preferentially triggering local drug release in the acidic tumor microenvironment. In EC, such systems can selectively deliver immunomodulators or pathway inhibitors to the tumor tissue while protecting the adjacent normal endometrium, which is crucial for fertility management [76].

Enzyme-responsive biomaterials provide additional guarantees for biological specificity by leveraging the upregulated proteolytic activity in tumor or damaged tissues. Matrix metalloproteinases (MMPs) are often overexpressed in epithelial cells and play a crucial role in tumor invasion, matrix remodeling, and inflammatory responses. They have been widely used as endogenous triggers. By integrating peptide sequences that can be cleaved by specific proteases into the main chain of polymers or cross-linkers, water gels and nanocarriers that can selectively degrade the network in a proteinase-rich microenvironment can be designed, thereby effectively releasing drug payloads. Importantly, such systems can be designed to have time programmability: anti-tumor drugs are released when proteolytic activity is high, and then regenerative signals are released as the enzyme levels decline, thereby achieving sequential support for tumor control and tissue repair.

In addition to pH and proteolytic activity, redox-responsive and inflammatory-responsive materials further expand the adaptive functional library of intelligent platforms. Tumor tissues and treatment-induced damaged sites typically exhibit elevated levels of reactive oxygen species (ROS) and reducing agents (such as glutathione (GSH)). Redox-responsive nanoparticles usually contain cleavable linkages (including disulfide bonds), which remain stable during circulation but rapidly degrade in high GSH or ROS-rich environments, thereby releasing therapeutic payloads locally. Meanwhile, inflammatory-responsive materials can be designed to respond to pro-inflammatory cytokines or oxidative stress, dynamically regulating drug release as the inflammatory load fluctuates. In EC, this responsiveness may enable biomaterials to weaken cytotoxicity or immunomodulatory delivery after inflammation subsides, thereby promoting the transition from tumor suppression to endometrial regeneration.

More and more multi-stimulus-responsive systems integrate sensitivity to multiple endogenous cues - such as the combination of pH and proteolytic activity or redox gradients - to optimize spatial and temporal control. These hybrid platforms can distinguish subtle changes in biochemical environments and achieve graded or sequential release patterns matching the evolution of disease states or regeneration stages. This complexity is particularly important in EC, as tumor microenvironment characteristics vary within and between lesions and dynamically change during treatment. In addition to endogenous triggering factors, exogenous stimuli such as temperature, light, ultrasound, and magnetic fields can also be used to activate bio-materials on demand. For instance, photo-responsive linkers or photothermal components can achieve precise external regulation of the timing and location of drug release. Such methods are particularly suitable for intrauterine or locally accessible applications, as they can apply exogenous triggering factors with high spatial precision.

Collectively, the expanding repertoire of stimulus-responsive biomaterials provides a versatile toolkit for adaptive therapy in EC. By bridging physiological features of the tumor and injured tissue, such as acidity, protease expression, redox imbalance and inflammation, with engineered responsiveness, these platforms enable drug delivery systems that are both spatially discriminative and temporally sensitive. Importantly, stimulus-responsive materials offer a tangible interface through which AI-guided therapeutic decisions can be operationalized: predictive algorithms that infer tumor activity, immune status or regenerative phase can be coupled to materials preprogrammed to respond to corresponding microenvironmental cues. In this way, intelligent biomaterials function as translational actuators, converting digital decision-making into localized, biologically appropriate therapeutic actions and allowing treatment to evolve in synchrony with disease dynamics.

### 5.3 Real-world evidence as a catalyst for clinical maturation

The complexity of biomaterial-enabled, subtype-guided therapies poses challenges for conventional randomized clinical trials, which are often ill-suited to capture long-term functional outcomes and adaptive treatment pathways. Therefore, real world evidence (RWE) plays a key role in validating the efficacy, safety and implementation of treatments [85,86].

The longitudinal RWE, obtained from a clinical record system, electronic health record and pregnancy outcome database, reveals persistence of treatment, reproductive success and long-term adverse events after the trial period. These data are particularly important for fertility preservation strategies, as pregnancy outcomes and offspring health can take years to manifest. In particular, RWE can inform iterative optimization of ai models and biomaterial design, creating a feedback loop between clinical practice and technological development. By combining real-world data with controlled trials, the approach supports the construction of learning health systems capable of continuously evolving with therapeutic innovation.

An integrated framework of artificial intelligence based decision making mechanisms, intelligent biomaterials and real world evidence is building the next generation of treatments for EC. This evolution has allowed the field to move beyond static multimodal combinations towards individualized adaptive systems integrating tumor biology, reproductive objectives and clinical reality. As these elements mature and harmonize, it is planned to redefine precision oncology for endometry cancer in a biologically based and deeply patient-centered manner.

## 6 Conclusions

**Fig. 7:**
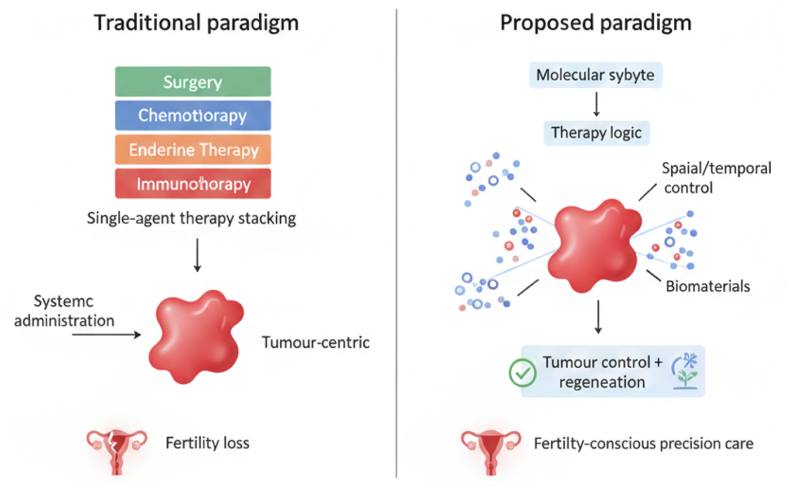
**A paradigm shift in integrated treatment of EC based on subtypes and fed by biomaterials.** Traditional models of treatment of EC focus mainly on therapeutic means and rely on empirical combined protocols of surgery, chemotherapy, endocrine therapy or immunotherapy, with limited consideration of molecular heterogeneity, dynamic microenvironment or long-term uterine function. The framework proposed in this review marks a shift towards an integrated subtype-driven concept powered by biomaterials. Molecular stratification guides therapeutic logic; Biomaterial platforms provide spatial, temporal and biological control of drug delivery; Regeneration objectives are integrated in parallel with oncological parameters. This integrated approach promotes uterine repair and fertility preservation while coordinating and regulating the immune, endocrine and metabolic pathways. By redefining biomaterial as an enabling infrastructure rather than a passive vector, the paradigm advances systematic strategies to harmonize tumor biology, patient-specific goals, and adaptive treatments. This integration provides a precise, sustainable, and fertility conscious treatment pathway for EC.

The clinical and biological complexities of ec are increasingly challenging traditional treatment approaches, of which monotherapy is the core. Its significant molecular heterogeneity, the interweaving of immune-endocrine-metabolic regulation, and the unique clinical needs for fertility preservation all make single therapies or empirical multi-faceted combination therapies ineffective. In this context, the strategies discussed in this review advocate for a fundamental rethinking of the conception, integration, and implementation of treatment for endometriosis (Fig. 7).

This review positions biomaterials as an enabling infrastructure for coordinated treatment, rather than an isolated technological advancement. The biomaterial platform provides space, time, and biological regulatory capabilities, enabling immunotherapy, endocrine regulation, and metabolic targeting to be precisely aligned within a subtype-oriented framework. Therefore, multimodal treatment shifts from simple additive operations to biological coordinated interventions - these interventions can respond dynamically based on tumor subtypes, microenvironment characteristics, and treatment stages. This coordinating function is particularly important in the treatment of EC that focuses on fertility, as the timing, location and sequence of anti-tumor and regenerative interventions must be actively managed rather than being randomly combined based on experience.

The key lies in this integration logic not only covering tumor eradication but also incorporating uterine repair and regeneration as a common primary treatment goal. For patients seeking fertility preservation, treatment success cannot be based solely on oncological endpoints; The restoration of uterine structure and function must also be taken into account. Inclusively incorporating reproductive expectations from the outset, a decisive departure from traditional methods and highlights the paradigm shift proposed in this paper. Looking to the future, the integration of molecular stratification technology, intelligent biomaterials, and data-driven decision-making provides new pathways for adaptive individualized diagnosis and treatment. The future of EC is not about further expanding the treatment toolbox, but about optimizing the decision-making system that determines how, when, and for which populations these tools are applied. To achieve this vision, interdisciplinary collaboration, regulatory innovation, and the continuous generation of real-world evidence are required.

In conclusion, this review redefines the biomaterial-enabled strategy as a catalyst for the reconfiguration of the treatment paradigm for EC, rather than an offshoot of drug delivery research. By advancing subtype-oriented, integrated, and fertility-aware treatment approaches, this study sketches a framework that enables the integration of precision oncology and regenerative medicine to fully meet the clinical needs of EC.

## CRediT authorship contribution statement

**Min Wang:** Conceptualization, Data curation, Formal analysis, Writing – original draft. **Cuihong Wang:** Data curation, Formal analysis, Writing – original draft. **Bing Xin:** Data curation, Formal analysis, Writing – original draft. **Lijie Zhao:** Data curation, Formal analysis, Writing – review & editing. **Benshuo Cai:** Conceptualization, Data curation, Formal analysis, Writing – original draft. **Yuheng Guan:** Writing – review & editing. **Lu Lu:** Conceptualization, Data curation, Formal analysis, Writing – original draft, Writing – review & editing. **Chong Zhang:** Conceptualization, Project administration, Supervision, Writing – original draft, Writing – review & editing.

## Declaration of competing interest

The authors declare that they have no known competing financial interests or personal relationships that could have appeared to influence the work reported in this paper.

## Data Availability

Data will be made available on request.
